# Effect of a comprehensive nutrition education program on nutritional behavior and food security of female-headed households who receive welfare support in Zanjan Province, Iran

**DOI:** 10.1186/s12889-023-16478-x

**Published:** 2023-08-09

**Authors:** Jalal Hejazi, Majid Aminzare, Yasamin Ayatollahi, Mohammad Masoud Vakili, Hassan Hassanzadazar, Mehran Rahimlou

**Affiliations:** 1 Department of Nutrition, School of Medicine, Zanjan University of Medical Sciences, Mahdavi Blvd., Shahrak-E Karmandan, P.O.Box: 4513956111, Zanjan, Iran; 2 Social Determinants of Health Research Center, Zanjan University of Medical Sciences, Zanjan, Iran; 3 Department of Food Safety and Hygiene, School of Public Health, Zanjan University of Medical Sciences, Zanjan, Iran; 4 Department of Health Education & Health Promotion, Zanjan University of Medical Sciences, Zanjan, Iran

**Keywords:** Nutrition education, Food security, Food safety, Physical activity, Low-income families

## Abstract

**Background:**

In recent years, the food security and dietary quality of many Iranian families have deteriorated due to unprecedented inflation. Nutrition education programs can be an effective and inexpensive method to improve food quality and security. The present study aimed to investigate the effect of a comprehensive nutrition education program for low-income women who are heads of households and are covered by the Zanjan province’s welfare.

**Methods:**

The food security of 2600 female-headed households covered by the Welfare of Zanjan province was evaluated using a standard 6-item questionnaire. A total of 600 women with the highest food insecurity scores were selected for the comprehensive nutrition education program. The participants received six sessions of 1.5 h of courses about how to improve the quality of their diets and manage their budgets and be physically active. At the beginning of the study and one month after the completion of the intervention, the participants were asked to complete a questionnaire designed and validated by the investigators. The scores of each section before and after the intervention were compared using paired t-test method and *p* values ​​of < 0.05 were considered statistically significant.

**Results:**

The prevalence of severe food insecurity among female-headed households who receive welfare support in Abhar, Khodabandeh, and Zanjan cities was 59.5%, 75%, and 62%, respectively. A total of 505 participants successfully completed the courses. After completion of the educational intervention, diet quality, physical activity, budgeting, and food safety scores of the participants increased by 6%, 4%, 4%, and 5%, respectively, which were statistically significant (*p* < 0.001). However, no significant difference was observed in the food insecurity scores.

**Conclusion:**

The comprehensive nutrition education program without financial or nutritional support can have a small but significant impact on the improvement of the nutritional behaviors and dietary quality of low-income people.

## Introduction

Food insecurity and improper nutrition have a significant impact on people's health. Various studies have shown that children from families with food insecurity may suffer from multiple developmental and mental disorders and the prevalence of obesity and various infectious and chronic diseases are higher among these children [1, 2]. In adults, malnutrition is also associated with a variety of chronic diseases. Numerous studies have shown a link between improper nutrition and obesity, diabetes, high blood pressure, and cancer. Iron deficiency anemia and decreased bone density, and consequent osteoporosis due to insufficient calcium intake are also common findings among people with malnutrition [3–5].

During recent years and after the re-imposition of US sanctions, Iran's national currency was devaluated dramatically, and sharp inflation has occurred in the food market. Some major food groups, such as meat, fruits, and vegetables, have experienced an inflation rate of more than 300% [6]. Especially after the pandemic of covid-19 virus, many people lost their jobs, and the purchasing power of a large portion of society has diminished severely. This situation will undoubtedly affect the food security and nutritional behavior of these people. It may push them to have an unhealthy eating pattern such as excessive use of starchy foods and simple sugars or high-fat foods which have lower prices, and sometimes by paying subsidies to these items (such as white bread), their costs are kept low.

Economic status and income level are among the factors that cannot be easily changed. However, nutrition education is a low-cost method that can significantly improve diet quality and reduce the unwanted effects of poverty on health [7]. Nutrition education can also impact food insecurity via mediators like improved resource management skills. There are examples of successful nutrition education programs in developed countries. The Expanded Food and Nutrition Education Program (EFNEP) in the United States, which began in 1969 and is now available in all states, is one of these programs. This program covers all low-income people who receive food aid from the US government. In this 6 to 8 sessions education program, participants learn how to choose cheap and healthy foods, and they earn the necessary skills in purchasing and financial management [8]. The cost-benefits and effectiveness of such programs have been evaluated by several investigators [7, 9]. For example, in a study by Dollahite et al., the authors have concluded that for every 1$ spent on EFNEP, the US health system benefits 10$ [10].

Unlike developed countries, in developing countries, there are a scarce number of documented and successful national nutrition education programs. In Iran's current situation, the need for nutrition education programs like EFNEP, especially for vulnerable groups, is pronounced. In a recent study by Mortazavi et al., the investigators assessed the effect of a nutrition education program on the food insecurity of households in southeast Iran [11]. According to their results, the number of food-insecure households in the intervention group decreased by 22% which was statistically significant [11]. The present study aimed to examine the effect of a comprehensive nutrition education program on the nutritional behavior and food security of female-headed households who receive welfare support in Zanjan province, Iran.

## Methods

### Study population and design

This study was performed on low-income women who are heads of their households and are covered by the Welfare of 3 main cities of Zanjan province including Zanjan, Abhar, and Khodabandeh. All of these women are widowed or divorced and they don’t have any established jobs and receive minimal monthly financial assistance from the welfare based on their family dimensions.

In the descriptive phase of the present study, we used the census method and all the women who were covered by the welfare organization in the three cities were evaluated in terms of food insecurity. The USDA six-item short-form Food Security Survey Module is used to determine food security status in the last 12 months. The total number of affirmative responses was summed to determine food security status as food secure (0–1) or food insecure (2–6). Food insecurity was further divided into low food security (2–4) and very low food security (5–6) [12]. The Persian version questionnaire has been validated by a previous study [13]. Based on the results of the questionnaire, 600 women with the highest food insecurity scores (5 or 6 out of 6) were recruited by convenience sampling method to participate in the education program. This group was chosen because they were the most food insecure and economically vulnerable segment of the population and if the program is successful in this segment of the population, for sure other segments will benefit from these kinds of education programs.

The education program consisted of 6 sessions, each lasting 1.5 h per week. All participants were asked to complete the Food Behavior Questionnaire, which was developed and validated by the investigators at enrollment. Participants received comprehensive nutrition education on topics such as healthy diet characteristics, budget-friendly meal preparation, shopping skills, financial management, and food safety. The content of the program was delivered through various methods, including short speeches, PowerPoint slides, an educational booklet, group discussions, films, and question–answer sessions. The contents were based on EFNEP. However, in the development of the program, the cultural, socioeconomic, and lifestyle features of the study population were considered.

After the completion of the 6-session program, there was a one-month gap before the participants were asked to complete the questionnaire again. This gap allowed us to assess the impact of the education program on the participants' nutritional behavior and food security over time.

Each participant received a 300000 rials gift card ( 2.5 $, during the implementation of the study) as a transportation assistance fee at the last session of the courses. The study protocol was approved by the ethical committee of Zanjan University of Medical Sciences (IR.ZUMS.REC.1399.193).

### Development and validation of food behavior questionnaire

The initial formulation of the questionnaire items was performed using the EFNEP Food Behavior and Physical Activity Questionnaire and extensive research in foreign and domestic information sources and taking into account the culture, eating habits, and social characteristics of our participants. After removing similar items, 22 items remained as the final items in the questionnaire. Based on the questionnaire developed by Murray et al. [14], there were 5 content domains in our questionnaire: diet quality (7 questions), food safety (4 questions), food insecurity (4 questions), food resource management (4 questions), and physical activity (3 questions). The response options for each question were based on a Likert scale. Each question was scored with higher scores indicating the greater frequency of the behavior in question. Positive and negative points were considered for positive and negative behaviors, respectively. The maximum score for the diet quality section was 24, for physical activity was 22, for food safety was 17, for food insecurity was 12, and for food resource management was 30.

The validity of the questionnaire was evaluated and analyzed based on the opinions of 10 members of the panel of experts, including specialists in health education, nutrition, food hygiene, and psychology. Two quantitative indicators of content validity ratio (CVR) and content validity index (CVI) were measured. Sequence, appropriateness to cultural and social values, and the need to remove or suggest the addition of new items were considered. According to Lawshe’s.

table Minimum value of CVR above 0.62 was deemed to be acceptable for each question [15]. According to our results, the CVR for all of the 22 questions was above the minimum value. Also, the CVI for each question completed by experts was above 0.79, so the questionnaire's validity was confirmed [15].

To evaluate the questionnaire's reliability, Cronbach's alpha value was calculated for 20 samples, which was equal to 0.857. Therefore, the reliability of the tool was also confirmed. The instrument's stability was assessed by the test–retest method at 15-day intervals by a group of 30 women from the target group (they were not included in the final study).

### Statistical analysis

Quantitative data are reported as mean ± standard deviation and qualitative data as frequency (%). The paired t-test method was used to compare the behavior change. The statistical analyses were performed using IBM-SPSS software version 22.

## Results

All female-headed households covered by welfare in Zanjan, Khodabande, and Abhar cities (*n* = 2600), were asked to complete the 6-item food security questionnaire. Of which 2054 participants completed the questionnaire. Based on the results, The participants who scored five or six were classified as the most severe food insecure group, and the ones who scored 0–1 were classified as food secure. The number of participants and the prevalence of severe food insecurity in each city is presented in Table 1.

**Table 1:** The prevalence of severe food insecurity among female-headed households covered by welfare in Zanjan Province

City	All Female-Headed Households^a^	People who completed the food insecurity questionnaire	People with severe food insecurity
Zanjan	1300	1121	696 (62%)
Khodabande	900	676	505 (75%)
Abhar	400	257	153 (59.5%)

^a^The numbers were rounded to hundreds due to the quota of the program

As can be seen, the prevalence of severe food insecurity is the highest in Khodabande (75%), the most underprivileged city in Zanjan province.

Among the participants with the most severe food insecurity, 600 women were recruited for the nutrition education program (450 from Zanjan, 100 from Khodabande, and 50 from Abhar). Of these, 505 individuals attended all the meetings and answered the questionnaires, that were included in the final analysis. The characteristics of these participants are presented in Table 2.

**Table 2:** Characteristics of people participating in the educational intervention at the base time (*n* = 600)

	**Mean**	**Standard Deviation**	**Minimum**	Maximum
Age (Year)	43.62	10.037	12	**86**
Family size	2.75	1.258	1	**8**
Education Level	Frequency (%)			
Illiterate	156 (30.9)			
Elementary	121 (24)			
High school	189 (37.4)			
Higher than high school	18 (3.6)			

As is shown, most of the participants had elementary or high school education.

All participants completed the Food Behaviors Questionnaire at baseline and one month after completion of the education program. The corresponding scores of diet quality, food safety, food insecurity, food resource management, and physical activity before and after the intervention, are presented in Table 3.

**Table 3:** Score related to diet quality, physical activity, food safety, food insecurity, and food resource management in the training group before and after the intervention (*n* = 505)

	**Before Intervention**	**After Intervention**	***p***
Diet Quality	5.07 ± 3.454	6.51 ± 3.793	**<**0.001
Physical Activity	6.62 ± 2.988	7.56 ± 3.256	**<**0.001
Food Safety	10.36 ± 2.968	11.23 ± 2.186	**<**0.001
Food Insecurity	8.41 ± 2.889	8.43 ± 2.884	0.829
Food resource management	20.58 ± 5.614	21.84 ± 5.172	**<**0.001

As seen, a significant improvement in the quality of diet, physical activity, food safety, and food resource management scores was observed after the intervention; however, there were no significant differences in the food insecurity scores before and after the education program.

Comparing the obtained scores with the maximum possible scores, it can be concluded that the participants in the present study are in an unfavorable state in terms of diet quality and physical activity, and in a favorable state in terms of food safety and food resource management. After the intervention, in total, there was a 6% increase in the food quality score, 4% in the physical activity score, 5% in the food safety score, and 4% in the food resource management scores.

Tables 4, 5 and 6, show the results of the educational intervention based on each city.

**Table 4:** Scores related to diet quality, physical activity, food safety, food insecurity, and financial management in the training group before and after the intervention in Zanjan City (*n* = 355)

	**Before Intervention**	**After Intervention**	***p***
Diet Quality	5.32 ± 3.669	6.35 ± 4.123	**<**0.001
Physical Activity	6.74 ± 3.311	7.37 ± 3.588	**<**0.001
Food Safety	10.96 ± 3.022	11.66 ± 2.266	**<**0.001
Food Insecurity	7.54 ± 2.669	7.58 ± 2.661	0.728
Food resource management	22.05 ± 5.732	22.23 ± 5.549	0.564

**Table 5:** Scores related to diet quality, physical activity, food safety, food insecurity, and financial management in the training group before and after the intervention in Khodabande City (*n* = 100)

	**Before Intervention**	**After Intervention**	***p***
Diet Quality	4.98 ± 2.825	6.45 ± 3.083	**<**0.001
Physical Activity	6.45 ± 2.100	7.27 ± 2.083	**<**0.001
Food Safety	8.04 ± 1.693	9.46 ± 1.123	**<**0.001
Food Insecurity	11.45 ± 2.076	11.55 ± 1.755	0.466
Food resource management	17.58 ± 3.217	20.68 ± 3.536	**<**0.001

**Table 6:** Scores Related to diet quality, physical activity, food safety, food insecurity, and financial management in the training group before and after the intervention in Abhar City (*n* = 50)

	**Before Intervention**	**After Intervention**	***p***
Diet Quality	3.50 ± 2.517	7.78 ± 2.023	**<**0.001
Physical Activity	6.16 ± 1.822	9.52 ± 1.693	**<**0.001
Food Safety	10.70 ± 2.261	11.70 ± 1.249	**<**0.001
Food Insecurity	8.48 ± 1.147	8.18 ± 1.746	0.145
Food resource management	16.12 ± 3.414	21.44 ± 4.841	**<**0.001

Comparing the effectiveness of the education program between different cities, it can be seen that in terms of the quality of diet, physical activity, and food resource management scores, Abhar had the highest rate of improvement, with 18%, 15%, and 18% increase, respectively. In terms of food safety score, the most increase was observed in Khodabande (8%).

## Discussion

In the present study, the comprehensive nutrition education program had positive effects in the four areas of diet quality, physical activity, food safety, and food resource management, however, no significant improvement was observed in the food insecurity score.

Numerous studies from all over the world in line with our findings have shown that nutrition education programs can be effective in improving diet quality, budgeting, food safety, and physical activity.

In a study by Srivastava and colleagues, the investigators conducted a comprehensive food and nutrition education program for low-income families of Tulare [16]. Two hundred and thirty-nine families were enrolled in their nine-week education program. After the intervention completion, significant improvements were observed in all the studied areas. Eighty percent of the participants improved the ways they manage their food resources. Also, improvement was observed in diet quality, food safety-related issues, and physical activities in 94%, 85%, and 55% of participants, respectively.

In another study, Farrell and colleagues presented 80 lessons to 80 EFNEP participants in eight sessions. The study results showed that food security among participants increased from 31% at the beginning of the study to 71% (*P* < 0.022). Participants prepared healthier food and ate more fruits and vegetables [17]. Auld et al. evaluated the effectiveness of the EFNEP program and observed its beneficial effects on physical activity (*P* < 0.001) [18].

Koszewski et al. examined the effect of EFNEP on participants 6 months after graduation to see the long-term impact of the program on the behaviors of the participants [19]. The results showed that 25% of participants were able to improve and maintain their behaviors. Physical activity and the use of grocery shopping, and financial management were among the improved items. Also, in food safety and the correct way of defrosting and storing food, positive results were observed. The authors suggested that more research is needed to prove the long-term benefits of the program on behavior changes.

However, in the present study, our nutrition education program was not effective in improving food insecurity scores and also the magnitude of improvement in the scores of other areas was relatively small. In accordance with our findings, some previous studies showed that the researchers did not see the expected effects of their education program on improving food security. For example, in a study by Miller et al., the researchers concluded that educational intervention would be more effective if nutrition education for low-income people were combined with food or financial aid [20]. Considering that most of our participants in the training sessions stated that the most crucial concern of them in achieving a healthy eating plan is the high cost of living and low income, the provision of food and financial assistance by the government or NGOs along with nutrition education programs can be an effective strategy for the improvement of food security in the population.

Moreover, in our study, about 100 participants did not complete the intervention courses and were excluded from the study. The cost of commuting, busy work, and lack of motivation were the most important reasons for not completing the course by these participants. In a nutrition education program by Omiuchen on the elderly population, many older adults did not participate in the program due to a lack of financial assistance. Also, many older people could not participate in the program due to transportation problems. The researchers concluded that financial aid is needed in addition to education and that barriers to accessing food for the elderly should be minimized [21].

Dickin et al. showed a link between parental behavior and children's eating habits and physical activities. Parental education can be a good part of promoting healthy eating and family activities [22]. In our study, it seems that the selection of females who are the head of their households as the target group for education is a good choice because modifying eating or physical activity-related behaviors in mothers can improve the whole family's lifestyle. However, in the present study, the actions of other family members were not investigated.

Along with numerous strengths, our study has encountered some limitations. The study was conducted in only three cities of the Zanjan province, which may not be representative of the entire population of female-headed households in Iran. This study only included women who were covered by the welfare system and did not have established jobs, which may limit the generalizability of the findings to other low-income populations. The study relied on self-reported data, which may have been subject to bias or inaccuracies. The study did not assess the long-term effects of the nutrition education program beyond one month after completion, and the study did not include a control group, which makes it difficult to determine whether the observed improvements were solely due to the intervention or other factors.

## Conclusion

The comprehensive nutrition education program can be an efficient and low-cost method for the improvement of diet quality, physical activity, food safety, and food resource management. However, the most successful nutrition education programs in the world, such as EFNEP or SNAP-ed, are the ones which in addition to providing nutrition education, also provide appropriate food assistance for low-income people, and the need for such aid, especially in the current situation of Iran, seems to be necessary.

## Data Availability

The datasets used and/or analyzed during the current study are available from the corresponding author upon reasonable request.
